# Plant Stem Cells in Cosmetic Industry

**DOI:** 10.3390/plants14030433

**Published:** 2025-02-02

**Authors:** Vassiliki Gardiki, Panagoula Pavlou, Angeliki Siamidi, Spyridon Papageorgiou, Apostolos Papadopoulos, Kriton Iakovou, Athanasia Varvaresou

**Affiliations:** 1 Section of Aesthetics and Cosmetic Science, Department of Biomedical Sciences, School of Health and Care Sciences, University of West Attica, 28 Ag. Spyridonos Str., Panepistimioupolis Egaleo Park, GR-12243 Athens, Greece; vgardiki@uniwa.gr (V.G.); ppavlou@uniwa.gr (P.P.); spapage@uniwa.gr (S.P.); apapadopoulos@uniwa.gr (A.P.); 2 Laboratory of Chemistry-Biochemistry-Cosmetic Science, Department of Biomedical Sciences, School of Health and Care Sciences, University of West Attica, 28 Ag. Spyridonos Str., Panepistimioupolis Egaleo Park, GR-12243 Athens, Greece; 3 Section of Pharmaceutical Technology, Department of Pharmacy, School of Health Sciences, National and Kapodistrian University of Athens, GR-15784 Athens, Greece; asiamidi@pham.uoa.gr; 4 Ministry of Health, 17 Aristotelous Str., GR-10433 Athens, Greece; kritoniakovou@outlook.com.gr

**Keywords:** plant cells cultures, cosmetic industry, skincare solutions

## Abstract

It is interesting to note that some of the most lucrative commercial products available today are derived from plant cell cultures. Apple, grape, ginger, rice, and other plant stem cells have been successfully and extensively utilized in cosmetic preparations all over the world. The advantages of plant cell suspensions over field-grown complete plants, which exhibit developmental stages of growth, plant age, and organ-specific differences, include sustainability, lack of pesticide residues, and independence from climate fluctuations. The procedure of extracting and purifying physiologically active compounds from plant cell cultures is significantly streamlined because of the possibility that these chemicals may be released into the intercellular gaps or wasted media through the cell walls and membrane. Upon downstream processing from the cells, the released chemicals exhibit minimal losses and a high degree of purity. Overall, the practical interest is in creating high-quality, sustainable, and innovative skincare solutions that meet both consumer needs and environmental concerns while driving the cosmetic industry toward more advanced biotechnological approaches. Our review intends to show the advantages of plant stem cells in cosmetic preparations.

## 1. Introduction

Cosmetics have been impacted by biotechnology in a number of ways. In order to create active compounds that can reduce the aging process, biotechnology is a good alternative approach. Recent research has focused on the special qualities of plant stem cells, both in the creation of novel cosmetics and in the investigation of the effects of these extracts on skin. Under stressful conditions, plants have a strong system for tissue regeneration. The plants’ regenerative activities include both the creation of new plants and the repair of damaged tissue. The Austrian botanist Gottlieb Haberlandt was the first to explain the process of callus formation from differentiated adult plant cells in 1902 [1]. He proposed that each plant cell had the capacity to rejuvenate the entire plant. Studies reveal that auxins are responsible for producing roots from a callus, whereas cytokines are in charge of producing stems [2]. The plant stem cells are grouped into niches, called meristems. These are classified into two main categories; the primary and the secondary meristems. The apical meristem (stem and root growth cone), the intercalary (insert) meristem and the germ meristem belong to the primary category, while the lateral ones-cambium (smear) and phellogen and the traumatic (callus) one belong to the secondary category. The negative reversible loop mechanism between gene expression products, i.e., WUSCHEL (WUS) and CLAVATA3 (CLV3) proteins, is one of the many variables that regulate the proliferation and differentiation of plant stem cells in the sprout apical meristem. The process that transforms somatic cells back into stem cells is carried out by the WUS protein [3].

An undifferentiated cell known as a stem cell could self-renew in order to divide or to give rise to multiple specialized cell types. Since most somatic cells have a limited life cycle, the stem cells’ ability to regenerate damaged somatic cells is essential for maintaining tissue homeostasis in numerous species. Because of their potential uses in aging research and regenerative medicine, there is an increasing attention in comprehending the process behind stem cells’ self-renewal and differentiation. Numerous beneficial cosmetic effects are attributed to plant stem cells, including: extending the lifespan of fibroblasts and encouraging their activity (e.g., *Gardenia jasminoides*, *Oryza sativa*); improving epidermis’ flexibility (e.g., *Capsicum annuum*, *Symphytum officinale*, *Opuntia* spp.); controlling cell division (e.g., *Lotus japonicus*, *Oryza sativa*); repairing epidermic damaged cells (e.g., *Opuntia* spp., *Panax ginseng*); initiating DNA cells’ repair, preventing oxidative stress (e.g., *Lycopersicon esculentum*, *Rubus ideaus*, *Citrus limon*); and providing UV protection (e.g., *Opuntia ficus indica*, *Dolichos biflorus*) [4].

**Figure 1:**
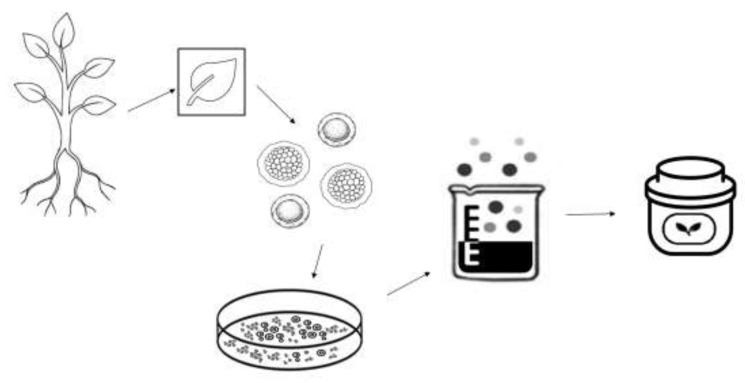
Plant stem cell processes leading to cosmetic preparations.

As a result, biotechnology and plant cell culture technology are one of the main interests of cosmetic research and development for new products in order to get beyond industry, consumer, and legal restrictions. Slow development, seasonal harvest, varying active concentrations from plant to plant and harvest to harvest, and the presence of poisonous metabolites have all hindered the usage of plants of cosmetic significance. Plant cell culture techniques have been demonstrated to solve these significant challenges in the creation of cosmetic items. This technology uses a variety of intricate techniques to guarantee that plant cells, tissues, or organs grow in an environment that contains nutrients free of microbes (Figure 1). This method makes it possible to synthesize biologically active compounds found in plants that are either difficult to obtain by chemical synthesis or not often found in the natural environment. Plant stem cell extracts that are produced using this technology are currently utilized to make cosmetics for both everyday use and professional use. Examples include pigments like safflower and saflorin from *Carthamus tincorius* and whitening agents like arbutin from *Catharanthus roseus* (rose periwinkle). Choosing the right plant material, sterilizing it, inducing callus, and subculturing it on commercially available Murashige and Skoog plant tissue culture medium are some of the processes in the extraction process. The cell line with the highest biomass production and the quickest doubling time can be chosen. High-pressure homogenization can be used to fully break up the suspended cells and liberate the active components from an established suspension culture. For improved topical distribution as a cosmetic product, the extracted plant stem cells can be encapsulated in different carrier systems [5].

Summarizing, biotechnology is being used to create active compounds that reduce aging, with a focus on plant stem cells. These have regenerative properties that can benefit skin health. Plants have a strong tissue regeneration system, which includes repairing damaged tissue and creating new plants. Plant stem cells are linked to various cosmetic benefits, such as extending fibroblast lifespan, improving skin flexibility, controlling cell division, repairing skin damage, and protecting against UV damage and oxidative stress. Using plants for cosmetic purposes has faced challenges like slow growth, seasonal variability, and toxic metabolites. These issues have been overcome by plant cell culture technology, which allows for the consistent production of active ingredients. This technology involves growing plant cells, tissues, or organs in a controlled environment, enabling the synthesis of valuable compounds. It is used to produce cosmetics with biologically active compounds that are difficult to obtain through chemical synthesis. The aim of this study is to explore the impact of biotechnology on cosmetics, particularly through plant stem cells, and investigate how plant stem cells contribute to skin care, aging research, and regenerative medicine. What are the challenges in using plant-based ingredients in cosmetics, and how does plant cell culture technology address these issues?

## 2. Methodology

The search strategy used in this review to identify the relevant literature includes specific search databases, keywords, and search filters applied. The keywords used in this review reflect the main topic of the industrial applications of plant stem cells in cosmetics. They are the following: Plant stem cells in cosmetics’’, “Plant stem cells in antiaging cosmetics”, “Antioxidant activities of extracts of plant stem cells”. The keywords include broad and specific terms related to plant stem cells applied to the development and efficacy of cosmetic products. Fifty review articles were downloaded. Since phytoconstituents and biotechnology have many applications in the research of Cosmetic Science, our target was to emphasize the current status in the cosmetic industry. Hence, we excluded the review articles that were relevant with plant stem cell extracts with potential application in cosmetics. The study design was based on review publications and there were cited articles with the main criteria being the use of plant stem cells in the cosmetic industry. The search period was between 2010 August 2024. The search engines were Pub Med, Scopus and Google Scholar. Thirty-eight review articles were finally studied. Specific research papers were also examined.

## 3. Cosmetic Industry Worldwide Using Plant Stem Cell Processes

Nowadays, a lot of pharmaceutical and cosmetic companies use bioreactors to create cutting-edge products with remarkable qualities. In vitro cell culture is used in the micropropagation process (also known as microreproduction) to replicate plant stem cells for cosmetic purposes. Choosing the plant part (leaf, fruit, or root) is the first step. The plant is then cut, and the extracted stem cells are transferred to agar plates to trigger the formation of callus. In a liquid environment, cells grow. Large-scale cell cultures employ specialized bioreactors because they guarantee regulated culture conditions. High pressure is applied to the developed stem cells, followed by the digestion of the cell membrane and the release of its contents onto the substrate. Unhwa Corp. has patented an anti-aging/antioxidant composition that contains chemicals as an active ingredient produced from a plant stem cell line derived from *Ginseng cambium* [6]. The product possesses antioxidant action that prevents reactive oxygen species—the primary cause of skin aging—from being produced by exposure to UV light. Zhang YC et al. explored the antioxidant activities of leaf extracts from different aged ginseng samples. Three different in vitro radical scavenging assays were applied, including scavenging capacity against 2,2-diphenyl-1-picrylhydrazyl radical (DPPH), 2,2′-azinobis-(3-ethylbenzthiazoline-6-sulphonate), free radical-scavenging capacity (ABTS) and hydroxyl radical-scavenging activity (HRSA). The antioxidant activity results revealed that the leaf samples from samples grown longer exhibit higher antioxidant activities, resulting from the increased accumulation of ginsenosides. [7]. It is also worth mentioning the *Ajuga repta* extract TEUPOL 10P or 50P. Other examples include cell suspension bioreactor cultures of *Haberlea rhodopensis*, *Rosa damascena,* and *Calendula officinalis*, as well as the utilization of bioreactors for the culture of stems by Mibelle Biochemistry [8]. Specifically, in 2008, Mibelle AG Biochemistry (Switzerland), a pioneer in the synthesis of plant stem cells for the cosmetic sector, created liposomes containing apple stem cells from the rare Swiss apple variety *Uttwiler Spätlauber* (PhytoCellTec^TM^ *Malus domestica*). The anti-wrinkle efficacy of PhytoCellTec *Malus domestica* was demonstrated in a clinical trial over 4 weeks with 20 subjects. A cream with 2% PhytoCellTec^TM^ *Malus domestica* was applied twice daily to the crow’s feet area. Wrinkle depth was analyzed with the PRIMOS system after 2 and 4 weeks. Digital photos of the crow’s feet area were taken at the beginning and the end of the study. The PhytoCellTecTM *Malus domestica* cream was found to significantly reduce wrinkle depth after two and after four weeks, by 8% and 15%, respectively [9]. Additionally, this company has developed plant stem cell extracts for the cosmetics sector from *Argania spinosa* (PhytoCellTec^TM^ Argan, Switzerland), *Saponaria pumila* (PhytoCellTec^TM^ nunatak^®®^, Buchs, Switzerland), and *Vitis vinifera* (PhytoCellTec^TM^ Solar Vitis, Switzerland). These products are presented in the form of extract suspensions.

An Italian team successfully produced *Syringa vulgaris* plant cells by cultivating plant tissues in vitro under aseptic conditions in growing containers that were treated with particular plant growth regulators [10]. Cell suspensions obtained from calluses underwent an aqueous extraction. Significant levels of verbascoside and isoverbascoside were detected by HPLC profiling. Strong antioxidant and scavenging action against free radicals was demonstrated by the extracts obtained from cell suspension. Furthermore, the produced extracts’ ability to inhibit lipoxygenase and 5-alpha reductase gave them excellent anti-hair loss qualities. Strong anti-tyrosinase activity and noticeable skin-whitening qualities were also demonstrated by the produced extracts [11].

Breton, De Lacharriere and Martin (2001) [12] produced undifferentiated cells of iris plants (*Iris germanica, Iris pallida* and *Iris florentina*). The produced cells were used to create novel cosmetic/pharmaceutical compositions containing an effective amount of at least one extract of at least one *Iridaceae*, for loosening and/or relaxing the cutaneous and/or subcutaneous tissue, especially for treating (reducing or eliminating) normal and small (fine) skin wrinkles [12].

French innovators created cosmetic products employing *Leontopodium alpinum* cells derived from in vitro cell cultures. The inventors published the invention as a patent in 2016 [13]. Specifically, in order to restore the homeostasis of the cells in aged skin and boost their metabolic and energetic activity, the invention offers the use of undifferentiated or undifferentiated plant cells of *Leontopodium alpinium* generated by in vitro cell culture as a non-therapeutic cosmetic treatment. A tensor and smoothing restorative effect is achieved with this treatment for aging skin, especially pronounced on the sagging neck skin and tear trough.

Ringenbach et al. produced cosmetic preparations using undifferentiated *Marrubium vulgare* cells as a raw material. In 2016, the patent was registered. Plant cells obtained through the in vitro cell culture procedure were used to create an active cosmetic component with the ability to improve the overall condition, look, and appendages of skin—more specifically, pore tightening and skin imperfections [14].

In 2016, an Italian team, Tito et al., created an active ingredient in cosmetic formulations. The use of somatic embryos from the plant species of *Citrus limon*, *Lotus japonicus,* and *Rosa gardenia* is the main emphasis of the invention covered by this patent. Effects of the aqueous, ethanol, and sugars/peptides extracts derived from *Lotus japonicus* somatic embryos were investigated on the gene expression of GDF11, SIRT1, and SIRT6. For the aqueous and ethanolic extracts, concentrations ranging from 0.05% to 0.0004% were tested, while for the mixture of peptides and sugars, concentrations from 0.01% to 0.00008% were used. The mixture of the three extracts of *Lotus japonicus* somatic embryos was more effective in activating GDF11 than the peptide/sugar extract alone, thus inducing a stronger response in the anti-aging and rejuvenation processes in skin cells [15].

Berry et al. extracted a dedifferentiated stem cell culture from *Camellia sinensis* var. *assamica* to create a cosmetic product that can shield skin from drying out and/or prevent damage from UV radiation. In 2017, the created product received a patent. The effectiveness of the innovation was evaluated using adult human dermal fibroblasts. The produced tea extracts, according to the inventors, showed anti-inflammatory qualities, stopped skin cells from drying out, and shielded skin cells from UV light [16].

Using the technique of Plant Cell Biofactories, OLEA VITAE ^TM^ (Vytrus bioteck) was produced from plant stem cells of wild olive tree sprouts (*Olea europaea var. silvestris*). Olive trees are distinguished for their lifespan, vitality, and resistance to drought and extreme heat. In particular, the wild olive tree was selected because of its higher level of genetic variety. Through a guided process of cellular disorganization, the phytolipid fraction was produced and released from the interior of the membranes of plant stem cells. In this way, the first generation of biomimetic lipids from the membrane stem cells, called phytolipid fractions, was created (Phyto-Lipidic Fractions, PLF). The phytolipid fraction of wild olive stem cells (*Olea europaea var. sylvestris*) OLEA VITAE ^TM^ can be characterized as a cellular oil, which mimics the action of cellular lipids, protects and intervenes in mitochondrial function, increases the available energy of skin cells, and boosts the production of skin structural proteins. The acceptance of the above claims regarding the properties of OLEA VITAE leads to the conclusion that this active substance has significant anti-wrinkle, firming, and rejuvenating properties.

## 4. Studies About Antioxidant and Anti-Aging Activity of Stem Cell Extracts

Studies have shown that kinetin, a cytokinin that is present in high concentrations in the stem cells of fruits like raspberries and citrus fruits, is one of the most potent inhibitors of the aging process of human cells. As a naturally occurring antioxidant, kinetin shields proteins and nucleic acids against oxidation and other harm. This substance functions in two ways. It can entail generating complex compounds with copper (II) ions that activate superoxide dismutase, the essential enzyme in the body’s antioxidation barrier, or stimulating the synthesis of regenerative enzymes, which eliminate altered bases from the DNA chain and offer defense against free radicals and oxidative stress. By preventing the formation of 8-oxo-dG, an oxidative indicator of genetic material damage that is produced as a result of the Fenton reaction, kinetin safeguards the cell DNA. As a natural growth hormone that enhances the function of the epidermis barrier, promotes the generation of keratinocytes, and decreases trans-epidermal water loss, kinetin is crucial for promoting skin stem cells. [17,18]. Cytokinins (CKs) are involved in many processes throughout plant growth and development. However, the precise roles of CKs have been difficult to analyze, mainly because (1) many of the classic CK responses are also controlled by other hormones (auxin, abscisic acid, or ethylene) or environmental stimuli (light or carbon/nitrogen source) and (2) the development of most of the physiological responses is slow (a few hours to days). [19,20].

The fact that tomatoes are high in antioxidant compounds, including lycopene, ascorbic acid, flavonoids, vitamin E, and phenolic acids, tomato-cultured stem cells seem to be promising in shielding skin from heavy metal toxicity. Furthermore, phytochelatins and other metal-chelating substances were present in tomato stem cell extract. Strong metal-binding proteins called phytochelatins, which are found in tomatoes, prevent heavy metal-induced collagen deterioration by blocking the action of collagenase. Additionally, it has been discovered that stem cells obtained from *Coffea bengalensis* cultures encourage skin cell regeneration by energizing fibroblasts to produce collagen. According to these studies, plant stem cells maintain remarkable anti-aging qualities and encourage fibroblasts to produce collagen, promote skin renewal, and fix damaged DNA] [8].

Anti-aging properties are also found in glycerin extracts from stem cells acquired from ginger (*Zingiber officinale*) leaf cell extracts, obtained by Naolys company (France). These properties were confirmed with a clinical study of 22 women. After 28 days of use, the skin structure was improved, pores reduced in size by 50%, and a matting effect (gloss reduction by 15% and sebum reduction by 19%) was observed after just six days of application. The in vitro results showed an increase in the synthesis of elastin and fibers and a reduction in the sebum production rate. In addition, Naolys also offers a glycerin extract from stem cells of *Iris pallida* (All Even Sweet iris), *Olea europaea* (All Fiber Booster Olive tree), *Hibiscus rosa* (All Fiber Booster Chinese hibiscus), and *Camellia sinensis* (All Fiber Booster Green tea), which also show anti-aging effects on the skin [21].

Phenylpropanoid glycosides, including verbascoside, isoverbascoside, leucosceptoside A, and martinoside, are linked to the antioxidant qualities of *Buddleja Davidii* stem cell culture [22]. Leaf cell culture extract from *Syringa vulgaris* is another abundant source of phenylpropanoid chemicals, particularly isoverbascoside [10]. According to Tito et al. [23], the tomato stem extract contains a high concentration of the flavonoids and phenolic acids present in tomato fruits. They discovered that the tomato cell extract had a higher overall antioxidant activity than the tomato fruit extract and that it included more rutin, coumaric, protocatechuic, and chlorogenic acids than the tomato fruit. Barbulova et al. discovered that a raspberry stem cell extract has strong antioxidant activity. The most common polyphenolic components analyzed were ferulic acid and quercetin rhamnoside. [24]. According to Bazylak and Gryn’s research, a number of plant stem cell extracts have antioxidant activity. Stem cell extracts of paper mulberry (*Brussonetia kazinoki*), grape (*Vitis vinifera*), magnolsi (*Magnolia sieboldii*), green tea (*Camelia sinensis*), white ginseng (*Panax ginseng*), and hydroponically cultured ginseng were tested for total polyphenolic content and DPPH radical scavenging. Although all the examined extracts exhibited strong antioxidant properties, the white ginseng and green tea stem cell extracts were the most successful [4,25].

Vichit W. and Saewan N. induced plant stem cells from the seeds of three rice varieties (Munpu, Hommali 105 and Niawdum) and investigated their ability to prevent aging. Vitamin E homologues, γ-oryzanols, anthocyanins, phenolic acids, and procyanidins are found in rice (*Oryza sativa* L.) and have been shown to have positive health effects, including lowering the formation of atherosclerotic plaque, inhibiting aldose reductase activity, lowering hyperlipidemia, and reducing the growth of cancer cells. Depending on the bioactive pigments found in the seed grain’s aleurone layer, pigmented rice can be light brown, reddish, or black. Black rice’s primary bioactive ingredient is cyanidin-3-glucoside, while procyanidin, catechin, and tannin have also been identified. Usually, pigmented rice extract outperforms non-pigmented rice extract in terms of nutritional quality and antioxidant activity, as it contains more phenolics in addition to pigment compounds. Inducing callus development significantly increased the rice seeds’ antioxidant (DPPH, PFRAP and SOD) and anti-aging (keratinocyte proliferation, anti-inflammatory, anti-collagenase and anti-tyrosinase) properties. The rice callus extracts appeared to have potent antioxidant and anti-aging activities, especially Munpu rice. Thus, rice callus extracts—especially pigmented rice—are a different natural source that has a lot of potential for use as anti-aging and antioxidant active ingredients in cosmetics, functional foods, and pharmaceuticals [26].

Red rice callus was cultivated from rice seeds and extracted using ethanol by the same team. Twenty-eight volunteers, ranging in age from thirty to fifty-five, were used to test the anti-aging effects of the induced rice callus extract in solution form. According to the study’s findings, when used topically, a product that contains 5% Thai red rice stem cell extract has anti-aging properties. Compared to baseline, statistical improvements were shown in its capacity to lower the melanin level and increase the aging skin’s flexibility and moisture content [27].

Rice stem cells were induced in vitamin-rich Chu N6 media using embryos of peeled, mature rice seeds. The well-induced three-week-old rice stem cells were exposed to plasma, cultured for a day, and then extracted to produce RSCE. Under conditions of oxidative stress, 0.1 mg/mL of RSCE after 3 min of plasma treatment showed quickly rising Human Fibroblast (HFB) cell proliferation rates in comparison to untreated RSCE, and the total phenolic content in 3 min of plasma-treated RSCE was increased by about 1.5 times in comparison to the control. According to this study, plasma therapy can increase cell viability by reducing cell damage and stimulating cell growth. The expression of genes associated with collagen synthesis protein and the proliferation of HFB after oxidative stress treatment, which is used to evaluate the efficacy of skin regeneration in plasma-treated RSCE, significantly increased by about four times in plasma-treated RSCE compared to untreated RSCE [28]. According to a clinical study performed by Gardiki V. et al., the application of a plant stem cell extract of *Olea europaea* incorporated into a cosmetic cream resulted in the decrease in erythema and hyperpigmentation caused after Nd:YAG 1064 nm laser epilation. In the same study, skin elasticity by means of the biophysical parameters measured by Cutometer statistically improved in comparison with the placebo cream. Skin biopsies showed that regenerative activity might be attributed to the extract [29].

## 5. Stem Cells in Skin Regeneration and Wound Healing

The effects of several fruit extracts from *Rhus coriaria* on the recovery of skin wounds have been documented. Gabr and Alghadir studied the aqueous extract derived from *R. coriaria* fruits, which contains hydrolyzable gallic acid, tannins, myricetin, and quercetin as a topical application. Results indicated that this formulation could accelerate the process of wound healing of both infected and uninfected rat skin. This inflammatory response regulation could be due to the increase in matrix myeloperoxidase and metalloproteinase 8 activity. Using a unique in vivo model, the application of a hydrogel containing an ethanolic extract from *R. coriaria* fruits to rat wounds accelerated the healing process by encouraging the synthesis of hydroxyproline and nitric oxide [30].

According to Yu Jin Won et al., extracts from *Rosa damascena* stem cells (RSCs) have several positive benefits on the skin. They postulated that exosome-like particles (RSCEs) may be released by RSCs in culture and that these particles may have a biological purpose in skin-related cells. After purchasing RSC culture supernatant, tangential flow filtration was used to carry out the conventional exosome separation procedure. While a high quantity of the crude supernatant caused extensive cell death, the RSCEs were shown to be non-toxic to human dermal papilla cells. Additionally, the RSCEs improved the closure of the scratch assay and the proliferation of human dermal fibroblasts, while the crude supernatant had no such impact. Additionally, in a dose-dependent manner, the RSCEs decrease the quantity of melanin in cultivated melanocytes and the amount of IL-6 generated by Raw264.7 cells activated by LPS. These findings demonstrate that RSC-released RSCEs containing proteins and miRNA have a variety of biological roles in skin-related tests, including fibroblast development and melanin concentration in melanocytes. Combined with the anti-inflammatory function of the RSCEs, they possess the right qualities to be helpful in esthetic medicine for enhancing the quality of skin [31].

Through a number of in vitro and in vivo tests, Cho, W.K. et al. assessed *Leontopodium alpinum* (Edelweiss) Callus Culture Extract (LACCE) as a natural cosmetic material, indicating its potent effects on the enhancement of skin and facial tissues. The molecular mechanism in human keratinocyte cells’ reaction to LACCE was specifically identified by RNA-Seq-based transcriptome analysis. In order to provide a skin barrier, which is supported by genes necessary for programmed cell death, LACCE up-regulated the gene involved in keratinization and cornification. LACCE treatment down-regulated a variety of stress-induced genes while up-regulating a large number of developmental process positive regulators. When combined, these findings showed that LACCE is a component of cosmeceuticals, or anti-aging cosmetics [32].

Guidoni M. et al. showed that stem cells from the *Coffee canephora* plant contain bioactive substances that have anti-inflammatory and tissue-repairing properties. They created a liposomal stem cell extract formulation from *C. canephora* leaves (LSCECC) and examined its potential to support dynamic tissue repair mechanisms. Atomic force microscopy, transmission electron microscopy, and the dynamic light scattering technique were used to develop and characterize the liposome cream. The LSCECC formulation, or vehicle control, was applied topically every day to the excisional full-thickness skin wound model. Five rats from each group were put down on days 2, 7, 14, and 21 following wounding, and biochemical and histological tests were used to assess the rates of wound closure and re-epithelialization. When compared to vehicle control, LSCECC produced faster re-epithelialization and showed a significant decrease in wound area of 36.4, 42.4, and 87.5% after 7, 10, and 14 days, respectively. Wounds treated with LSCECC showed increased granular tissue and a healthy inflammatory response, which was mediated by an increase in IL-10 and a decrease in pro-inflammatory cytokines like TNF-α and IL-6. Additionally, wounds treated with LSCECC exhibited enhanced scar tissue quality because of increased transforming growth factor-beta and vascular endothelial growth factor, as well as increased collagen fiber deposition and organization at the wound site. Thus, LSCECC is a promising bioactive extract to support and enhance wound healing, extracellular matrix production, inflammatory response modulation, and neovascularization [33].

The main wound healing stem cell extracts are summarized in Table 1.

## 6. Interaction of Plant Stem Cells with UV

Secondary metabolites produced from biotechnologically produced meristem cells are highly effective in protecting human skin in vivo and keratinocytes in culture from UV radiation damage. Their molecular structure, which consists of condensed aromatic 5- and 6-carbon rings with numerous OH-groups, has led to them being considered effective photoprotective factors. They can effectively absorb UV-A and UV-B irradiation (sunscreen qualities) thanks to their structure without promoting further photochemical processes [34]. Because the glycosyl moiety increases photo-stability, many glycosylated metabolites are resistant to UV-A light destruction. Active ingredients generated from meristem cells have shown remarkable efficacy in shielding human skin fibroblasts and blood vessel endothelial cells from bacterial and UV-induced cell damage. In the presence of active chemicals from meristem plant cells, human keratinocytes subjected to bacterial lipopolysaccharides or pro-inflammatory cytokines produced significantly lower levels of reactive oxygen species and harmful cytokines. Their preventive effect was similar to that of non-steroidal anti-inflammatory medications and topical corticosteroids [35].

In biological systems, plant cell cultures exposed to suitable elicitors seem to be a special and useful source of low molecular weight modulators of the oxidative state (direct and indirect antioxidants, targeted free radical scavengers). These tiny molecular molecules may be the best modulators of the cutaneous oxidative state since they are accessible and biocompatible with human skin cells. Grapevine berries (*Vitis vinifera*), for instance, are widely regarded as a valuable source of phenolic acids, catechins, and anthocyanins, such as peonidin 3-glucoside, cyanidin 3-glucoside, malvidin 3-glucoside, cyanidin 3-p coumaroyl glucoside, etc., which are direct antioxidants and powerful free radical scavengers [36].

It was discovered that applying a grape stem cell extract (PhytoCellTec Solar Vitis- INCI: *Vitis vinifera* Fruit Cell Extract, a product of Mibelle Biochemistry, Buchs, Switzerland) to epidermal stem cells increased their colony formation efficiency (CFE) by 86%. Furthermore, it was shown that this extract offered UV protection. The grape stem cell extract was reported to neutralize a UV exposure that caused a 50% reduction in CFE in untreated control cells. In a placebo-controlled clinical investigation, the grape stem cell extract was also used to create a sun lotion that decreased UV-induced erythema [37].

## 7. Hair Follicle Aging

The ability of apple tree stem cells, or *Uttwiler Spätlauberr* stem cells, to slow down skin aging the Mibelle AG Biochemistry firm examined the process of tissue atrophy. One insulated hair follicle section was placed on the growth nutrient (control sample) in the experiment, while the other was placed on the nutrition that had 0.2% of the stem cell extract added. Because they were in the anagenic phase and protected from skin fragments following a facelift, human hair follicles made an ideal model. When placed on growth nutrients, they were able to grow longer for an additional 14 days.

The insulated hair follicles were unable to proliferate after this time because of the lack of blood flow, as their cells either aged or underwent apoptosis. After 14 days of the experiment, it seemed that the follicles in the control sample had shrunk, while those on the nutrition containing the plant stem cell extract continued to grow until the eighteenth day [38].

## 8. What Are the Gaps in the Safe and Reliable Use of Stem Cells in Cosmetics, and Why Should These Problems Be Addressed?

Terminology is important when claims for cosmetics are made. For instance, it is important to know that when the word “plant stem cell” is used as an ingredient, it refers to the primitive cell extract. Many skin care firms are using stem cell technology to promote their products. Cosmetic companies really use stem cell extracts rather than live stem cells in their products, despite their claims to the contrary [5]. Plant stem cell cultures cannot be used in cosmetics [5].

Direct-to-consumer marketing of stem cell-derived products or stem cell-based procedures is practiced by thousands of companies worldwide. While some do include stem cells but are not yet clinically established or sufficiently regulated, others are incorrectly classified as stem cell-related. Because they are biological products, need to be handled very carefully. First and foremost, laboratories that prepare stem cells for the production of cell-derived products or whole cell utilization ought to be subject to stringent regulations. Second, for each desired goal, it is essential to define specific manufacturing processes, such as tissue source and collection, cell isolation, cell culture and manipulation, phenotypic profile, concentration or quantity of cells administered, and mode of administration. Thirdly, to verify safety and efficacy, sufficient clinical trials must be carried out for every application. It is essential that suppliers of a certain product or process do not injure their customers or engage in dishonest business practices by making unsubstantiated claims. Stem cell operations and products must adhere to truth-in-advertising regulations and the safety criteria set forth by the local Drug and Medical Devices Authorities. It is also important to note that stem cell cosmetics are not the only product of this insufficient control. A technique is commonly brought to market in the cosmetics business before undergoing sufficient clinical testing. This is due to the regulatory organizations in charge of monitoring being either unprepared or incapable of handling this quickly changing business. For the cosmetics sector to meet demand and maintain market share, it must provide the newest social media cosmetic fads, including unapproved or untested goods and processes. Thus, it is essential to educate customers on the prerequisites, the approval procedures, and any systemic flaws in cosmetic products or procedures. In order for customers to report any suspected malpractices and educate themselves on the product or process they plan to buy, this is crucial [39,40,41].

## 9. Conclusions

In recent years, the cosmetics sector has gained global attention due to increased customer involvement and the integration of biotechnology. Biotechnological methods have enabled the production of a wide range of active cosmetic ingredients, leading to the development of safe and effective formulations for cosmetic and esthetic treatments. Biotechnology’s ability to create organically based products with minimal environmental impact has driven this trend, with advancements in industrial and environmental biotechnology improving manufacturing processes, reducing greenhouse gas emissions, and eliminating harmful chemical contaminants. Research has evolved from traditional fermentation to modern methods such as gene and recombinant DNA technologies, enabling the efficient synthesis of low-toxicity products, renewable bioenergy, and environmental monitoring techniques. Plant cell cultures have emerged as a powerful, adaptable method for producing high-quality extracts with specific skin care benefits. These extracts, cultivated in controlled environments, ensure sustainability and avoid harm to endangered plant species, making them increasingly popular in skin care and functional cosmetics. RNCOS Business Consultant Services’ most recent consumer and research reports predict that plant “stem” cell technology will propel the global cosmetics market forward because it offers new potential for product creation and scientific innovation. Although plant stem cell technology holds great potential for the cosmetics market, the high costs of production and maintenance remain a challenge. However, emerging biotechnological methods are expected to offer more cost-effective and environmentally friendly solutions in the near future.

## Figures and Tables

**Table 1:** Positive effects of plant extracts on skin wound healing.

Plant Extract	Study Focus	Key Findings	Mechanism/Effect
*Rhus coriaria* L. (Aqueous Extract) [33]	Topical application for wound healing in rats	Accelerated wound healing in both infected and uninfected skin. Increased matrix myeloperoxidase and metalloproteinase 8 activity.	Regulation of inflammatory response, enhancement of wound healing through increased enzymatic activity.
*Rosa damascena* Stem Cells (RSCEs) [34]	Effect of exosome-like particles (RSCEs) on skin-related cells	RSCEs improved fibroblast proliferation, scratch assay closure, reduced melanin, and decreased IL-6 in melanocytes and immune cells.	Fibroblast development, melanin regulation, anti-inflammatory properties.
*Leontopodium alpinum* (Edelweiss) Callus Culture Extract (LACCE) [35]	Natural cosmetic material for skin and facial tissue enhancement	LACCE upregulated genes involved in keratinization, cornification, and developmental processes. Downregulated stress-induced genes.	Promotes skin barrier, keratinization, and anti-aging effects.
*Coffee canephora* Stem Cells (LSCECC) [36]	Liposomal formulation for wound healing in rat models	LSCECC accelerated re-epithelialization, reduced wound area, and enhanced scar tissue quality. Increased IL-10 and decreased pro-inflammatory cytokines.	Supports tissue repair, reduces inflammation, enhances extracellular matrix production, and promotes neovascularization.

## Data Availability

All data generated during this study are included in this published article.
